# Machine learning based multi-modal prediction of future decline toward Alzheimer’s disease: An empirical study

**DOI:** 10.1371/journal.pone.0277322

**Published:** 2022-11-16

**Authors:** Batuhan K. Karaman, Elizabeth C. Mormino, Mert R. Sabuncu, for the Alzheimer’s Disease Neuroimaging Initiative

**Affiliations:** 1 School of Electrical and Computer Engineering, Cornell University and Cornell Tech, New York, NY, United States of America; 2 Department of Radiology, Weill Cornell Medicine, New York, NY, United States of America; 3 Department of Neurology and Neurological Sciences, Stanford University, Stanford, CA, United States of America; University of North Carolina at Chapel Hill, UNITED STATES

## Abstract

Alzheimer’s disease (AD) is a neurodegenerative condition that progresses over decades. Early detection of individuals at high risk of future progression toward AD is likely to be of critical significance for the successful treatment and/or prevention of this devastating disease. In this paper, we present an empirical study to characterize how predictable an individual subjects’ future AD trajectory is, several years in advance, based on rich multi-modal data, and using modern deep learning methods. Crucially, the machine learning strategy we propose can handle different future time horizons and can be trained with heterogeneous data that exhibit missingness and non-uniform follow-up visit times. Our experiments demonstrate that our strategy yields predictions that are more accurate than a model trained on a single time horizon (e.g. 3 years), which is common practice in prior literature. We also provide a comparison between linear and nonlinear models, verifying the well-established insight that the latter can offer a boost in performance. Our results also confirm that predicting future decline for cognitively normal (CN) individuals is more challenging than for individuals with mild cognitive impairment (MCI). Intriguingly, however, we discover that prediction accuracy decreases with increasing time horizon for CN subjects, but the trend is in the opposite direction for MCI subjects. Additionally, we quantify the contribution of different data types in prediction, which yields novel insights into the utility of different biomarkers. We find that molecular biomarkers are not as helpful for CN individuals as they are for MCI individuals, whereas magnetic resonance imaging biomarkers (hippocampus volume, specifically) offer a significant boost in prediction accuracy for CN individuals. Finally, we show how our model’s prediction reveals the evolution of individual-level progression risk over a five-year time horizon. Our code is available at https://github.com/batuhankmkaraman/mlbasedad.

## Introduction

Alzheimer’s Disease (AD) is the most common type of dementia among the elder population, accounting for nearly 70% of all dementia patients [1] and ranking as the seventh leading cause of death in the United States [2]. Many mechanisms in the development of AD have been uncovered by decades of experimental and clinical studies [3–5], but the jigsaw is still unsolved. In the realm of AD, public and private databases [6–10] serve as important resources for the application of machine learning algorithms that help characterize disease heterogeneity [11], guide therapy [12], and develop and evaluate potential treatments [13, 14]. An area where machine learning can play a crucial role is the prediction of future clinical decline at the individual level, which can inform clinical and other life decisions.

Clinically, in the context of Alzheimer’s disease, individuals are classically grouped into one of the following three stages: cognitively normal (CN), mild cognitive impairment (MCI), and AD dementia. MCI is considered a high-risk, transitionary stage between healthy aging and dementia. Future clinical decline toward dementia is considered to be more predictable in those with MCI than in CN individuals [15, 16]. As our results further corroborate, CN-to-MCI conversion is inherently a more difficult prediction problem than MCI-to-AD conversion. The vast majority of the published studies dealing with individual-level future decline predictions with machine learning focus on early detection of MCI-to-AD conversion [17].

There is growing literature showing factors, including certain biomarkers, that predict progression from CN to MCI [18, 19]. This work has converged to suggest that the risk associated with specific factors is relatively small, and requires a long follow-up to observe the effect. Furthermore, papers tackling the CN-to-MCI conversion prediction problem with machine learning have been relatively limited [20, 21]. Thus, there is a need to understand what combination of variables can yield accurate individual-level predictions; and whether the significance of the variables changes as a function of disease stage (e.g., CN or MCI at baseline).

Many prior studies have primarily focused on building models that predict future conversion within a specific time horizon, e.g., three years [22, 23]. Although some of these studies test their modeling strategy for variable follow-up years, they do this by training new models for each time horizon. Studies that utilize survival (event-time) models can, in theory, make predictions for any future time-point [24], yet, they need to make strong assumptions about the evolution of the underlying hazard function, which might potentially limit performance.

In this work, we rely on the neural network (deep learning) framework, which gives us the flexibility to explore the effect of various modeling choices, namely nonlinearity and predicting arbitrary time horizons, while holding other design parameters constant. We implement three different classifiers, two of which are trained to predict the clinical status (CN, MCI, or AD) at a single time point in the future. The first of these two models is linear and called the “Linear Single-year Model (LSM)”. The other one is nonlinear and referred to as the “Nonlinear Single-year Model (NSM)”. In the third model, we employ a nonlinear architecture and modify it to make it capable of predicting the clinical status at any time point in the future. We refer to this as the “Nonlinear Multi-year Model (NMM).” Comparing these models allow us to deduce the predictive importance of nonlinear models and accounting for different time horizons. For instance, our results verify that a linear model (LSM) can yield high-quality predictions for the relatively easy MCI-to-AD conversion prediction problem, whereas higher capacity nonlinear models are needed for making more accurate predictions about the more challenging task of predicting CN-to-MCI conversion.

Our analyses further offer some novel insights. In CN individuals, predicting who will convert to MCI within a year is easier than predicting for a 5-year time window. For individuals with MCI, however, the situation is reversed. It is harder to predict who will progress to AD in the shorter term. We train our models to handle arbitrary missingness patterns in the input data, which in turn, allows us to interrogate the predictive value of different data types. For example, our results suggest that the molecular biomarkers we consider in our study are very helpful in the MCI stage, but not as much in CN individuals. On the other hand, structural magnetic resonance imaging (MRI) biomarkers (hippocampus volume, specifically) offer a significant performance boost for CN-to-MCI conversion but not MCI-to-AD conversion. Finally, we present our model’s prediction of future conversion risk as a continuous function of time, which reveals different trajectories.

## Materials and methods

In this section; we first present the dataset, which was derived from the Alzheimer’s Disease Neuroimaging Initiative (ADNI) database [25]. We then discuss important implementation details that were critical in handling missingness, and unbalanced classes at different time points.

### Dataset

All participants used in this work are from the Alzheimer’s Disease Neuroimaging Initiative (ADNI) database. ADNI aims to evaluate the structure and function of the brain across different disease states and uses clinical measures and biomarkers to monitor disease progression. We select the participants who did not have clinical AD at the baseline (screening) visit and had at least one follow-up diagnosis within the next five years (n = 1404). We excluded the CN baseline participants who converted to AD in the next five years because they are very few (n = 6), the CN baseline participants who converted to a later stage but reverted back to an earlier stage (n = 23), and the MCI baseline participants who either were diagnosed as CN in a later follow-up year (n = 87) or converted to AD and reverted back (n = 18) since these subjects might have been diagnosed incorrectly at some point. Including these individuals did not alter our main conclusions. After the exclusions, we are left with 1404 participants. We note that all individuals were either CN or MCI at baseline. Table 1 lists summary statistics for the participants; including sex, age, number of years of education completed, count of Apolipoprotein E4 (APOE4) allele, Clinical Dementia Rating (CDR), and Mini Mental State Examination (MMSE) scores at baseline.

**Table 1:** Summary statistics of the participants at baseline.

	CN baseline (*n* = 615)	MCI baseline (*n* = 789)
Female/Male	335/280	324/465
Age (*yr*)	73.19 ± 6.18	73.46 ± 7.39
Education (*yr*)	16.51 ± 2.57	15.93 ± 2.81
APOE4 (0/1/2)	430/169/14	371/313/98
CDR	0.04 ± 0.13	1.55 ± 0.89
MMSE	29.11 ± 1.11	27.52 ± 1.82

Mean ± standard deviations are listed. APOE4 row represents the number of alleles.

A critical aspect of the data, as is common in many real-world longitudinal studies, is that there are missing follow-up visits, with imperfect timings, and many subjects drop out of the study before the planned completion. Table 2 shows the number of available subjects in each diagnostic group for annual follow-up visits. We note that Table 2 can be used to infer the number of subjects progressing from one stage to another during follow-up years of interest. In Table 2, and in all subsequent analyses, any subject who progressed (from CN to MCI, or from MCI to AD) before dropping out of the study was considered to remain MCI or AD until year 5. Non-converter subjects without a visit in a certain follow-up year are not used for either training or testing in that follow-up year. Some subject groups have different follow-up visit schedules. For example, in ADNI-2 and ADNI-3, CN baseline participants are only clinically evaluated every other follow-up year. Therefore years 1 and 3 have fewer CN diagnoses than years 2 and 4, respectively. ADNI includes a limited number of subjects who have been monitored for more than five years. However, due to the very limited number of visits beyond 5 years, we excluded these timepoints from our analyses.

**Table 2:** The number of available subjects in each diagnostic group for annual follow-up visits.

Follow-up year	CN baseline		MCI baseline	
	CN	MCI	MCI	AD
1	427	14	674	110
2	527	32	431	218
3	181	41	317	261
4	230	49	202	286
5	123	54	127	292

The follow-up diagnoses are not actually exactly 12 months apart. They have been rounded to the nearest time horizon in years.

### Input features

We use the clinical data and biomarkers collected at baseline as our input features. Clinical data includes subject demographics (age, gender, number of years of education completed, ethnicity, race, marital status), genotype (number of APOE4 alleles), clinical assessments (clinical dementia rating, or CDR; Activities of Daily Living, or FAQ; Everyday Cognition, or ECog), cognitive assessments (Mini-Mental State Exam, or MMSE; Alzheimer’s Disease Assessment Scale, or ADAS-Cog; Montreal Cognitive Assessment, or MoCA; Rey Auditory Verbal Learning Test Trials 1–6; Logical Memory Delayed Recall; Trail Making Test Part B; Digit Symbol Substitution, Digit and Trails B versions of Preclinical Alzheimer’s Cognitive Composite score [26, 27]), and the baseline diagnosis (CN or MCI). The biomarkers are Cerebrospinal Fluid (CSF) measurements [28, 29] (Amyloid-Beta 1–42; Total Tau, or T-Tau-; Phosphorylated Tau, or P-Tau), Magnetic Resonance Imaging (MRI) volume measurements [30, 31] (Ventricles; Hippocampus; WholeBrain; Entorhinal; Fusiform; MidTemp; Intracranial Volume, or ICV; all computed using the FreeSurfer software [32–51]), and Positron Emission Tomography (PET) standardized uptake value ratio (SUVR) scores (for following tracers: Fluorine-18-Fluorodeoxyglucose, or FDG; Florbetapir, or AV45; Pittsburgh Compound, or PIB) [52, 53]. We note that CSF, FDG, and PIB biomarkers are referred to as molecular biomarkers. Furthermore, we employ single, global PET SUVR measurements instead of regional values.

The regional volume measurements derived from the MRI scans were computed, quality controlled and publicly made available by UCSF researchers. In this pipeline, the images are processed to implement the following steps: Talairach transform computation, intensity normalization, skull stripping, creation of the white-matter and pial surfaces, segmentation of the gray and white matter volumetric regions of interest and creation of the cortical parcellation as described in [54]. Note that we input ICV as a separate feature, instead of normalizing other volumetric MRI measurements with it.

The ADNI study consists multiple phases (1, GO, 2, and 3). Each phase implemented a slightly different data acquisition protocol. Additionally, as mentioned above, follow-up data collection was also heterogeneous, with missing visits and non-uniform visit intervals. The degree of missingness for the different baseline data modalities is shown in Table 3 for the 1404 participants we use. Rather than dropping out the subjects with incomplete baseline data, we substitute placeholder values for their missing features and keep them in our dataset. Our substitution procedure consists of two parts. First, we record the binary missingness mask for the feature set. Each participant has their own binary missingness mask indicating what variable is observed or not for that particular individual. Then, following prior work [55], we perform mode substitution for missing categorical variables (sex, ethnicity, race, marital status, APOE4), and mean substitution for missing numerical variables. It is true that when the class labels are unbalanced, these substituted values will be biased by the values in the majority class. However, we would like to emphasize that we consider these substituted values as dummy placeholders. In other words, we use mode/mean values solely to make sure that the substituted values are within an appropriate range and thus the numerical optimization is not compromised. By concatenating the missingness mask to the feature vector, we expect the model to learn to treat these placeholders appropriately. To prevent any information leakage, we substitute the mode and mean values computed in the non-missing portion of the training set as placeholders for the missing values in training, validation, and test sets. We compute a single mode/mean value for each feature, weighing all non-missing values in the training set equally.

**Table 3:** The degree of missingness (%) in different baseline data modalities for two patient groups.

Data type	CN baseline	MCI baseline
Clinical assessments	27.87	37.51
Cognitive assessments	7.38	7.34
CSF	48.29	37.14
MRI	33.36	21.22
FDG	45.37	24.08
AV45	46.99	50.95
PIB	100	98.48

There is significant missingness in the biomarker data. Crucially, there is no CN baseline patient with an AV45 scan at baseline. Moreover, a substantially limited amount of MCI subjects have AV45 scans at baseline.

The categorical variables except the baseline diagnosis are one-hot encoded (i.e., are represented with dummy variables encoding presence) and numerical variables are z-score normalized in the last step of feature processing. We note that the z-score normalization is first performed on training data and mean and variance values are saved to be used for validation and test data later on, which is similar to what we do in the second step of the imputation procedure.

We have six discrete (categorical) variables, which are sex (encoded as a one-hot vector for either female or male), ethnicity (encoded as a one-hot vector for either Hispanic/Latino or not Hispanic/Latino), race (encoded as a one-hot vector for one of the following: Asian, Black, Hawaiian/Other PI, Indian/Alaskan, More than one, White), marital status (encoded as a one-hot vector for one of the following: divorced, married, never married, widowed), number of APOE4 alleles (encoded as 0, 1 or 2 copies of the E4 allele), and baseline diagnosis (a scalar that is 0 for CN and 1 for MCI). Real-valued variables are the number of years of education completed, clinical test scores, cognitive assessments, and biomarker values. All real-valued (numerical) features are scalars except for the clinical assessment of ECog, and the cognitive assessments of ADAS-Cog, and Rey Auditory Verbal Learning Test Trials 1–6, which are vectors with dimensionalities 14, 3, and 4, respectively. In total, we have 45 real-valued features. We note that we also compute a binary missingness mask for the input feature set. In the mask, there is no entry for the baseline diagnosis, since that variable has no missingness. Therefore, the binary missingness mask has a dimensionality of 50. Concatenating the categorical features, numerical features, and binary missingness mask yields a feature vector of length 113.

### Models

We are interested in the prediction of an individual’s future diagnostic status (CN, MCI, or AD) based on the input features at a baseline visit. A large number of studies have looked at this question as a classification problem, often at a single follow-up time, e.g., three years after the baseline. However, this formulation has two drawbacks. First, many subjects might drop out of the study before that follow-up time, which means these subjects cannot be used for training. Secondly, this approach groups together subjects who convert after the intended follow-up time, with those who are stable through the study. To distinguish these two subject groups, one would need to train a new model from scratch corresponding to a different follow-up time. An alternative approach that addresses these issues involves survival modeling [56]. However, these methods require strong assumptions about the underlying hazard function that can constrain the model’s performance.

**Fig 1:**
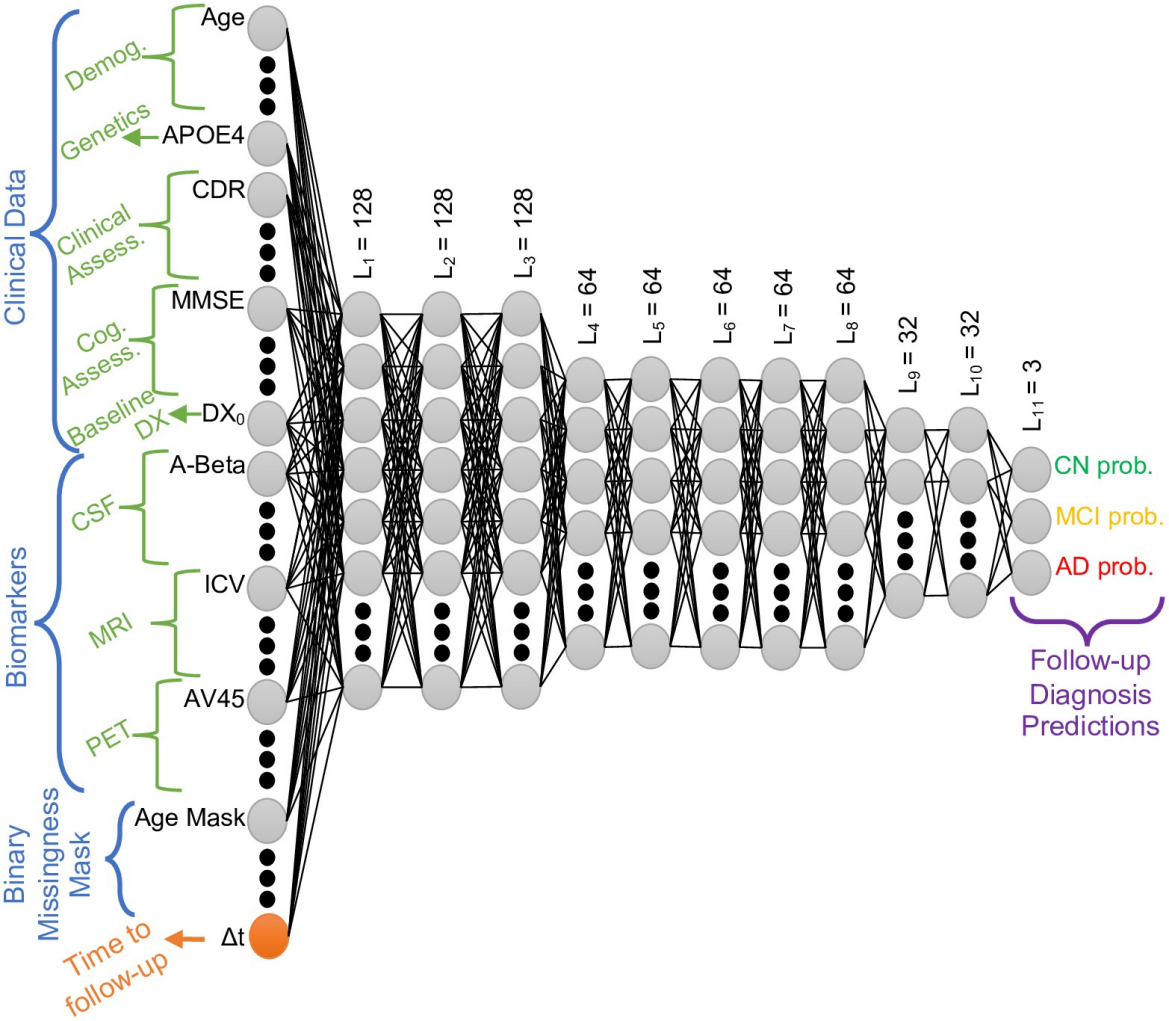
Feed-forward, fully-connected neural network architecture. Nonlinear models have rectified linear units (ReLU) between layers. Final layer implements a soft-max. L_l_: Number of neurons in layer l. Input features include following. Demog.: Deomographics. Clinical Assess.: Clinical Assessments. Cog. Assess.: Cognitive Assessments. Baseline DX: Baseline Diagnosis. prob.: probability.

All our models follow the neural network architecture template depicted in Fig 1, which was designed based on optimizing empirical performance on validation data in a single split. The output is a length-3 probability vector, computed by a soft-max layer, that corresponds to the individual’s CN, MCI, and AD probabilities at the future time point. In the single-year models, the prediction is for a fixed follow-up time and thus time-to-follow-up is not an input feature. Therefore, the single-year models have an input layer width of 113, and the input layer width of the multi-year model is 114. We train a separate single-year model for each time horizon. The linear single-year model, LSM, is made up of linear (fully connected) layers, whereas its nonlinear counterpart, NSM, contains nonlinear activation functions, which are elementwise rectified linear units, or ReLUs, between linear layers. The most flexible model, the nonlinear multi-year model, or NMM, accepts the time-to-follow-up (in months) as an input feature and computes the output corresponding to that input value. Thus, we train a single NMM for all follow-up times. The NMM can be viewed as a family of models, parameterized by the follow-up time. We note that all three models have roughly the same number of learnable parameters.

We also experimented with two alternate models. The alternative single-year linear model was a standard linear regression model, implemented as a single fully connected layer neural network, with L2 penalty (weight decay) on the weights (coefficients). This is equivalent to a ridge regression approach. As the results presented in the Supplementary Material demonstrate this model performs no better than the LSM model described above. The second alternative is a slight modification of the NMM model, where the input time-to-follow-up feature is encoded as the closest annual visit time. This model (results in S1 and S2 Tables) performed very close to the NMM model we present here.

### Loss function

We use categorical cross-entropy loss to train our neural networks:

$$\begin{array}{c} -\frac{1}{N}\sum_{i=1}^{N}\;\left\langle y_{i},\text{log}\left({\hat{y}}_{i}\right)\right\rangle \end{array}$$ (1)

where *N* is the number of training datapoints, 〈, 〉 is the inner product operator, *y*_*i*_ is the one-hot encoded vector for the ground truth label of sample *i* (i.e., is a dummy variable vector encoding presence), and ${\hat{y}}_{i}$ is the probability vector of the sample point *i* that is computed by the classifier. The expression in Eq (1) represents the average of the losses across the entire training dataset, which implies that each sample has the same weight. This is undesirable in unbalanced problems due to the fact that the contribution of the majority class to the loss function will be higher compared to the minority class.

As can be seen in Table 2, there are two types of imbalance we need to consider.

1. The distribution of class labels varies significantly over the years. The number of CN baseline participants who convert to MCI is smaller than the number of non-converters over the five-year period, but the relative difference shrinks in later years. For participants who are MCI at baseline the situation is more drastic. Those who convert to AD represent a small minority in the early years, yet MCI-to-AD converters are the majority at the 5-year mark.
2. The number of available clinical labels decreases with each follow-up year, since individuals drop out of the longitudinal study.

Naively using the loss term in Eq (1) would encourage certain types of errors. For example, the model would care less about accurately classifying CN-to-MCI converters, particularly in earlier years. This would affect all three of our models. The second imbalance factor, on the other hand, would exclusively impact the performance of NMM. With the loss in Eq (1), NMM would pay less attention to later follow-up years than earlier ones which means the performance of NMM would suffer in longer time horizons. We note that the LSM and NSM approaches are not affected by this because a separate model is trained for each follow-up year.

There are well-established ways of addressing imbalance issues. Under-sampling the majority class, over-sampling the minority class, and using re-weighted loss functions are the most popular options. In this work, we employ a loss re-weighting scheme. In this approach, the model is penalized more for an error in the minority class than an error in the majority class, using sample-level weights. The loss function we use in this work is

$$\begin{array}{c} \text{Loss}=-\frac{1}{N}\sum_{i=1}^{N}w_{i}\;\left\langle y_{i},\text{log}\left({\hat{y}}_{i}\right)\right\rangle \end{array}$$ (2)

where **w**_*i*_ is the weight of the sample point *i*. We propose a weighting scheme that accounts for both sources of imbalance we discussed above. Although converter CN baseline participants and non-converter MCI baseline participants belong to the same ground-truth class, we do not weigh those samples equally as they represent different prediction scenarios. Therefore, for a given follow-up year, we consider four possible categories of participants: CN baseline non-converters, CN baseline converters, MCI baseline non-converters, and MCI baseline converters. In the first step, we compute the weights for each category as one over the size of each group. In step 2, we scale these weights so that the total weight of each follow-up year is equal. By doing so, we are addressing the second imbalance.

In the single-year models, the ground-truth clinical status, e.g. for one year after the baseline, was the diagnosis made at the visit corresponding to that follow-time. We note that the timing of this visit is typically not exact and can deviate by several months. For example, a planned 1-year follow-up could have occurred around 15 months after the baseline visit. Thus, the ground-truth labels can be viewed as noisy. On the other hand, for the NMM model, since the follow-time is not fixed and is treated as an input variable (coded in months), the ground-truth label can be viewed as more accurate. That said, as we described above, we implemented a version of the NMM model that accepts the rounded annualized visit times as input (see Supplementary Material) and we observed no meaningful difference in results.

### Experimental details

We implemented a randomized, diagnosis stratified 80–20 split of the data into train-test sets. We repeated this 80–20 split 200 times and all presented results are averaged over these repeats. For each 80–20 split, we also conducted a 5-fold cross-validation on the train sets, where the validation loss was used for early stopping. For each cross-validation fold and modeling choice, we trained 5 different models with different random initializations. Thus, for each test case, the final prediction is computed as the average of the 25 model predictions (5 cross-validation and 5 random initializations each). For the NMM model, we ensure that all longitudinal follow-up data for a participant is in the same partition. All three of our models use the same data splits.

The model architecture and hyperparameter values are fixed for NMM and single-year models, with the only difference being the missing input neuron of Δt in LSM and NSM. These choices were manually determined based on inspecting the validation loss in a single split. The architecture, illustrated in Fig 1, has 3 hidden layers with a width of 128, followed by 5 hidden layers of width 64 and 2 hidden layers of width 32. We perform early stopping during training based on the validation loss. We employ an L2 penalty loss on the weights and biases, with a weight of 10^−6^ in each hidden layer. We implement dropout after each hidden layer with a rate of 0.2. Our optimizer is Adam with a learning rate of 10^−5^. We use the softmax activation function at the output layer.

In order to explore the influence of the network architecture on results, we conduct two more experiments where we use the same training strategy, activation functions and hyperparameters as NMM, however we modify the architecture that is illustrated in Fig 1. The first experiment uses an elementary 3-layer nonlinear architecture with fewer parameters than NMM. The second experiment is a computationally expensive practice where we optimize the nonlinear architecture via a grid search over depth and width hyperparameters in each one of the 200 train/test splits. In this experiment, each test set has its optimal architecture, identified by the hyperparameter values that yield the best performance on a validation set. Results of both experiments are presented in S1 Fig. We note that the overall trend and pattern of the NMM’s results hold consistent across these architectural choices. The 3-layer model’s performance is slightly worse and the optimized architecture results are the best, as expected. The 11-layer model we present in Fig 1 under-performs slightly compared to the optimized architectures. We note that the 11-layer model was manually designed to optimize performance in a single train/validation split.

We analyze prediction accuracy in two different patient groups: CN at baseline and MCI at baseline. Therefore, each result we show has two parts, one corresponding to the CN-to-MCI conversion task and the other to the MCI-to-AD conversion task. In each task, clinical conversion is considered a positive event. For example, for the CN-to-MCI conversion task, a true positive sample refers to the subject progressing to MCI and the model predicting this correctly. Accordingly, the true positive rate is defined as the ratio of true positive samples against all converter subjects, and the false positive rate is the ratio of false positives against all stable subjects.

Due to the heavily unbalanced nature of the data, which can be seen in Table 2, we use the receiver operating characteristic (ROC) curve to inspect the performance of our models. Although there are no subjects who convert from CN to AD or from MCI to CN in our dataset, we do not implement any mechanism to prevent our models from making such predictions. Therefore, both CN-to-MCI conversion and MCI-to-AD conversion tasks are multi-class problems for our models. There are two different types of ROC analyses for multi-class problems: one-versus-one analysis and one-versus-rest analysis. As we demonstrate with our results, our models capture the disease progression dynamics sufficiently that both analyses give nearly identical ROC curves. In order words, the predicted AD probability for CN baseline subjects and the predicted CN probability for MCI baseline subjects are close to 0. Thus, we only share one-vs-rest results where the positive class for CN-to-MCI conversion is MCI and for MCI-to-AD conversion it is AD.

The area under the ROC curve (ROC AUC) is a scalar that summarizes the overall performance of a classifier. Our data has a time horizon of five follow-up years and we evaluate against annual diagnoses. For each of our three models, we compute an ROC AUC value corresponding to each follow-up year and each baseline group (CN baseline and MCI baseline). Therefore, each model has five ROC AUC values associated with each baseline group. To statistically compare the ROC AUC values achieved by two different models, we implement a pairwise permutation testing strategy, yielding a p-value for the null hypothesis that the two models’ predictions are indistinguishable. Our test statistic is the difference between the mean ROC AUC values (averaged over the annual follow-ups) for the two models in a given 80–20 train-test split. We then average this over all 200 random splits of our data. To create the null distribution of the test statistic, we randomly permute (10^5^ times) the two models when computing the ROC AUC difference for each split. Finally, the normalized rank of the observed (unpermuted) test statistic value among all sorted (permuted) test statistic values yields the p-value, which we denote with *ρ*.

## Results

### Impact of modeling choices

In CN-to-MCI conversion, we observe that there is a substantial difference between the linear and nonlinear models. For example, for the 1-year follow-up, LSM yields 83.88% ROC AUC, whereas its nonlinear counterpart, NSM, achieves 88.73%. This difference remains stable over all follow-up years and is statistically significant (*ρ* < 0.0001). The multi-year training strategy, on the other hand, further boosts prediction accuracy. For instance, for the 1-year follow-up, NMM achieves an ROC AUC of 90.40%. The difference with the NSM model is consistent over the follow-up years and statistically significant (*ρ* = 0.0001). Finally, we note that for CN-to-MCI conversion, all models tend to achieve worse performance as the time-horizon increases. For instance, the best-performing NMM model suffers more than a 6% drop in ROC AUC between 1- and 5-year follow-up predictions. This result suggests that it is easier to predict who will convert from CN to MCI in the relatively short term, say within a year, than in the longer term, say within 5 years.

We notice that the performance of LSM fluctuates as a function of the time horizon. There are two local minima, one at 2- and another at 4-year follow-up. This is likely because those two years include a higher percentage of CN subjects, due to the study design of ADNI 2 and 3, as can be seen in Table 2. We see that this affects the performance of the nonlinear single-year model, NSM, too. However, for the NMM the issue is mitigated, which is likely because the multi-year model can leverage the data from the other follow-up years to “smooth out” its predictions.

**Fig 2:**
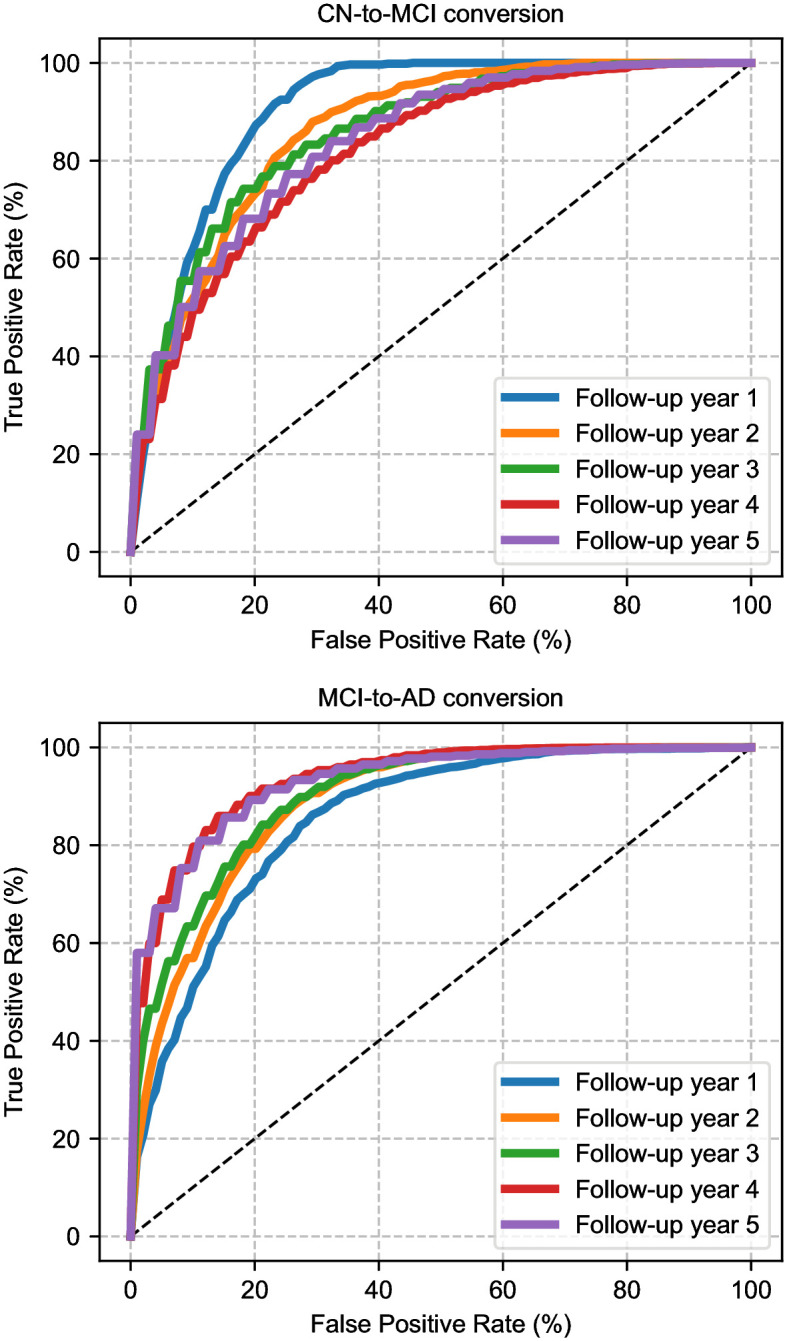
ROC curves of NMM for CN-to-MCI and MCI-to-AD conversion in five-year time horizon. Displayed are averages of 200 train-test splits.

In MCI-to-AD conversion, there is an overall diminished difference between the performance of the three models. For the single-year models, the linear and nonlinear counterparts are statistically indistinguishable (*ρ* = 0.3735). The multi-year model, on the other hand, offers a statistically significant (*ρ* = 0.0004 against LSM, *ρ* = 0.0014 against NSM), yet subtle boost in ROC AUC, specifically for 1- and 2-year follow-ups. In the remaining follow-up years, all three models achieve essentially the same performance level. The most striking observation from the MCI-to-AD conversion results is that prediction accuracy improves for later years, and there is a very consistent increase in ROC AUC values across all modeling choices. This indicates that it is relatively easier to predict who will convert from MCI to AD in the 4–5 year horizon compared to the 1–2 year horizon. Fig 2 shows corresponding ROC curves of NMM for each follow-up year and each patient group.

### Contribution of different biomarkers

As mentioned above, our models are capable of handling missing values in the input. This allows us to inspect the contribution of different data types to prediction accuracy. We perform this analysis on our best performing model, NMM, where we focus only on test participants with complete baseline data and systematically mask each input feature, treating it as missing.

Our baseline scenario is where only clinical data (CD) is available. Fig 4 shows the difference in AUC ROC (Δ AUC ROC) achieved with the utilization of additional biomarkers: FDG PET (a single global marker of sugar metabolism), CSF (global markers of tau and amyloid burden), AV45 PET (a single global marker of brain amyloid load), MRI volumetric measurements (markers of brain atrophy). For MRI, we consider two scenarios. First, we only use the value of hippocampus volume, normalized by the intracranial volume (ICV) (CD+ICV normalized Hippocampus size in Fig 4). In the second scenario, we use seven MRI-derived AD-associated biomarkers (CD+MRI in Fig 4). As a reference, we also show the results for including all available biomarkers in these test subjects that have complete baseline data (CD+All Biomarkers in Fig 4). We were not able to quantify the contribution of PIB PET, as only a very limited number of participants have PIB PET scans.

In CN-to-MCI conversion, molecular biomarkers (FDG, CSF, and AV45), by themselves, do not significantly improve performance over the baseline CD-only scenario, particularly beyond the 1-year follow-up (*ρ* = 0.1898 for CD+FDG, *ρ* = 0.2082 for CD+CSF, *ρ* = 0.3001 for CD+AV45). However, we observe a substantial accuracy boost when MRI data are available (*ρ* < 0.0001), much of which can be attributed to the hippocampus volume (*ρ* < 0.0001). All biomarkers combined achieve the highest ROC AUC values (*ρ* < 0.0001). The performance gain grows over the years, suggesting that additional biomarkers are more useful for making longer-term predictions.

Overall, the performance gain offered by additional biomarkers is relatively smaller for the easier MCI-to-AD conversion problem. Here, MRI markers add around 1% ROC AUC to the CD-only baseline. FDG consistently yields a greater boost than the MRI biomarkers in each follow-up year, which is in contrast to what we observe in CN-to-MCI conversion. Crucially, we find that hippocampus volume does not provide a statistically significant performance boost (*ρ* = 0.2412), while FDG and MRI markers improve the model performance subtly but significantly (*ρ* < 0.0001 for CD+FDG, *ρ* = 0.0024 for CD+MRI). Beyond year 1, CSF consistently outperforms AV45 (*ρ* < 0.0010 for CD+CSF, *ρ* = 0.0253 for CD+AV45), where the latter yields a boost on par with MRI. This highlights the potential importance of tau markers, particularly in the MCI stage. Overall, however, a striking observation is that the model that has access to all the biomarkers is substantially more accurate than a model with a single biomarker type.

### Disease progression risk predictions

Even though we consider the problem as a three-label classification task for a given follow-up time, the underlying process can be viewed as a continuous evolution of MCI and AD dementia risk [57]. Using our NMM model, we can compute a prediction for arbitrary time horizons for the test subjects and interpret the output probabilities as a longitudinal estimation of risk. The softmax outputs of the MCI channel for CN baseline participants and AD channel for the MCI baseline participants are shown in Figs 5 and 6, respectively. We average these values over test subjects who have the same conversion time profile.

For individuals who remain stable CN throughout the 5-year follow-up period, we observe that NMM’s MCI prediction is consistently less than 50%. Intriguingly, for those stable subjects who were last observed earlier, the predicted MCI probabilities tend to be higher. In fact, for stable CN subjects last seen before the end of year 2, average predicted MCI probabilities exceed 50% around the year 4 mark. We emphasize that the model has no access to follow-up information, as the only input is baseline data. For subjects who convert to MCI at year 1, average predicted MCI probabilities exceed 0.5 before the first annual follow-up visit. Similarly, for those who convert around the second year, the average predicted MCI probabilities exceed 0.5, between years one and two. One notable exception is the group of individuals who progress to MCI at the third-year visit. In this group, the NMM prediction is that MCI conversion will happen, on average, at around the 5-year mark.

For the MCI baseline subjects, we observe similar patterns. For the stable subjects, the predicted AD probabilities remain under 0.5 until the last follow-up visit. For MCI-to-AD converters, the average predicted AD probability exceeds 0.5 before the AD diagnosis, except for the subjects who convert at the 5-year follow-up, where the average predicted AD probability is slightly below 0.5 at the 5-year mark. On the other hand, when we examine the timing of the average predicted conversion, it seems to be less accurate than with CN baselines. In most scenarios (e.g. conversion at 2, 3, and 4 years), the average predicted AD probability exceeds 0.5 before the corresponding time interval. This suggests that the NMM’s predictions tend to estimate an earlier MCI-to-AD prediction than observed.

## Discussion

In this work, we present an empirical study to characterize how predictable an individual subject’s future AD-associated clinical trajectory is, several years in advance, based on rich multi-modal data, and using modern deep learning methods. We present a novel machine learning strategy that can handle variable follow-up time queries, missingness patterns, and unbalanced class labels in the data, to make accurate predictions about the future decline in CN and MCI baseline participants.

**Fig 3:**
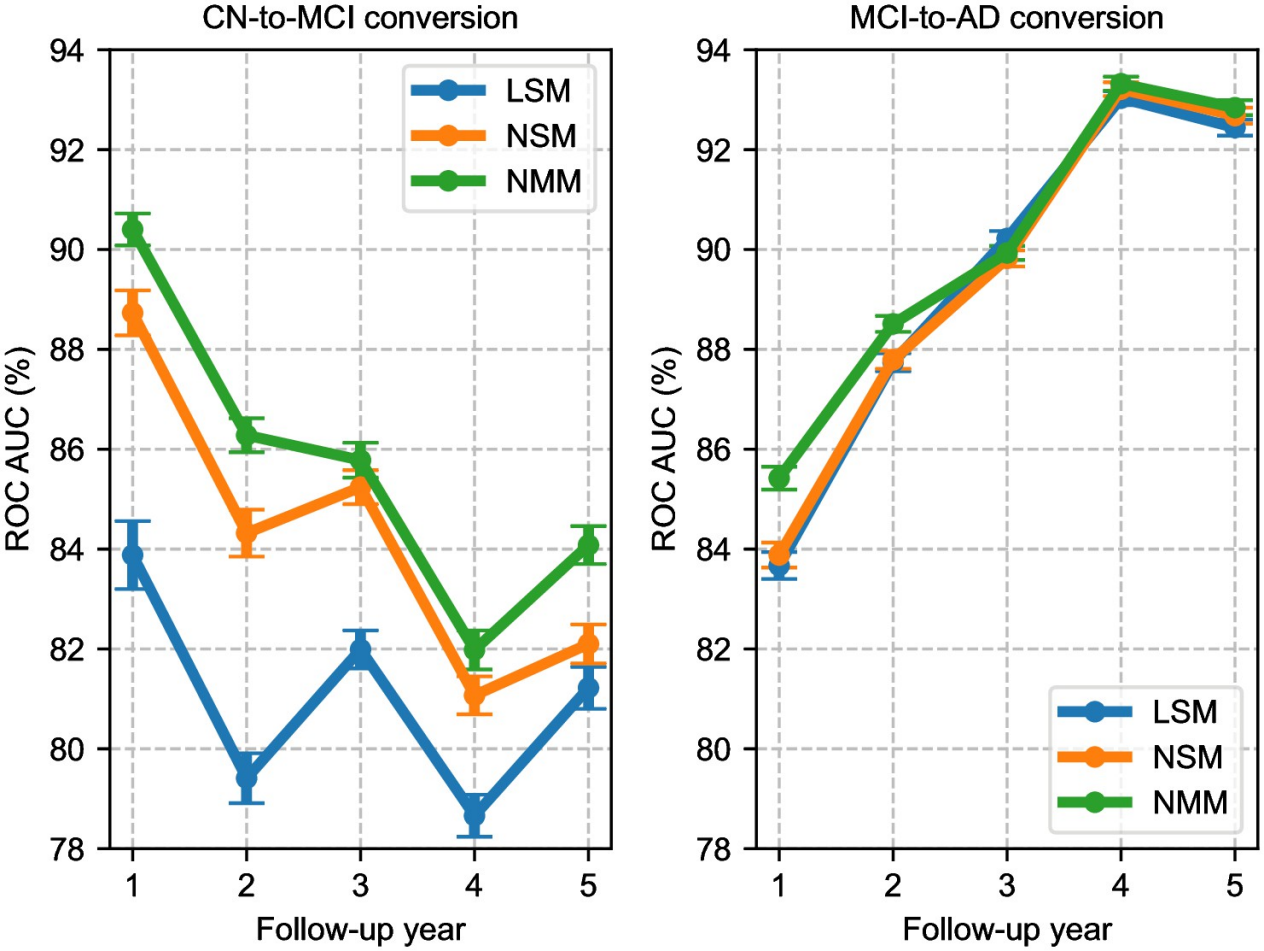
Predictive performance of different models for different follow-up years. ROC AUC values are averaged across 200 80–20 data splits. Error bars indicate the standard error across these splits. LSM, Linear Single-year Model; NSM, Nonlinear Single-year Model; NMM, Nonlinear Multi-year Model.

Comparing the prediction accuracy for CN-to-MCI and MCI-to-AD conversions in Fig 3, our results verify that the CN-to-MCI conversion prediction is a harder task than the MCI-to-AD conversion prediction. On the other hand, we also confirm that more sophisticated modeling, such as a nonlinear multi-year (NMM) architecture, offers a larger boost for the harder CN-to-MCI conversion prediction task. This verifies that there is a bigger gap in performance between what a relatively simple model can achieve and the upper bound of what is achievable (also known as the Bayes-optimal performance) in the harder problem of CN-to-MCI conversion.

Five years is a relatively short time window for studying CN-to-MCI conversion. On the other hand, in many real-world clinical scenarios, 5 years is a useful horizon to consider. Moreover, we note that at year 5, around 30% of the baseline CN subjects who remained in the study had converted to MCI, as we show in Table 2. Our analysis demonstrates that the prediction of CN-to-MCI conversion gets harder for distant time horizons, and we achieve higher accuracy for shorter time frames. This insight might be useful in detecting those CN subjects who might be on the cusp of developing MCI.

**Fig 4:**
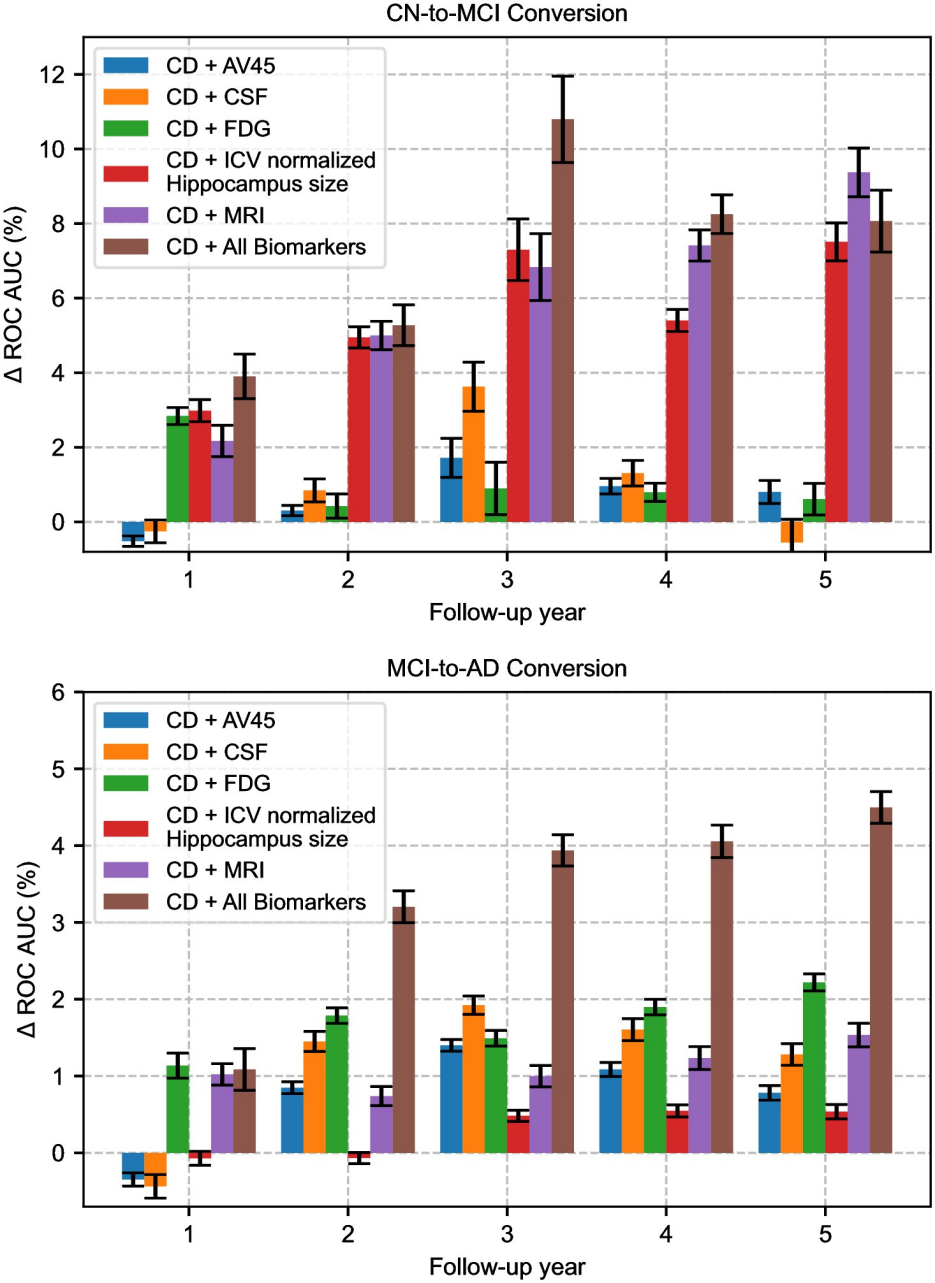
Δ ROC AUC values obtained with NMM by the addition of various biomarker combinations to the clinical data (participant demographics, clinical assessments, and cognitive assessments). ROC AUC values are averaged across 200 80–20 data splits. Error bars indicate the standard error across these splits. +: Used together. CD, Clinical data; AV45, Florbetapir PET; CSF, Cerebrospinal Fluid; FDG, Fluorine-18-Fluorodeoxyglucose PET; MRI, Magnetic Resonance Imaging; ICV, Intracranial Volume.

Despite the missingness in the data, Fig 4 suggests that NMM does not rely solely on a single modality. Additional biomarkers, in general, do not make the prediction performance worse. This finding is in parallel with the fact that multi-modal data, such as different MRI sequences and various PET tracers are often combined in the literature for predicting MCI-to-AD conversion [58–60]. However, our results also demonstrate that the predictive value of each additional biomarker can vary. For example, for CN baseline participants, although there is a substantial accuracy increase with the use of MRI; molecular biomarkers (CSF, FDG, and AV45) do not offer a significant boost beyond the first year horizon. For the prediction of MCI-to-AD conversion, however, the situation is different—molecular biomarkers offer a significant boost. Furthermore, using the different MRI biomarkers together seems to be much more helpful for predicting MCI-to-AD conversion, rather than relying on a single MRI biomarker, namely the ICV-normalized hippocampal volume. These results highlight the importance of characterizing the diagnostic and predictive utility of different data types, at different stages of the disease process.

One interpretation of the patterns of results we present in this study might be that amyloid or tau-associated biomarker changes have a relatively longer timecourse than MRI derived measurements, such as hippocampal volume. Furthermore, MRI markers may be less specific and reflect a multitude of effects that result in atrophy, particularly at later ages. Thus, MRI might predict more proximal decline from CN to MCI, but its utility will be less during the MCI stage, where tau/amyloid markers might offer some specific insights into the Alzheimer’s pathology dynamics that will play out over the next several years.

**Fig 5:**
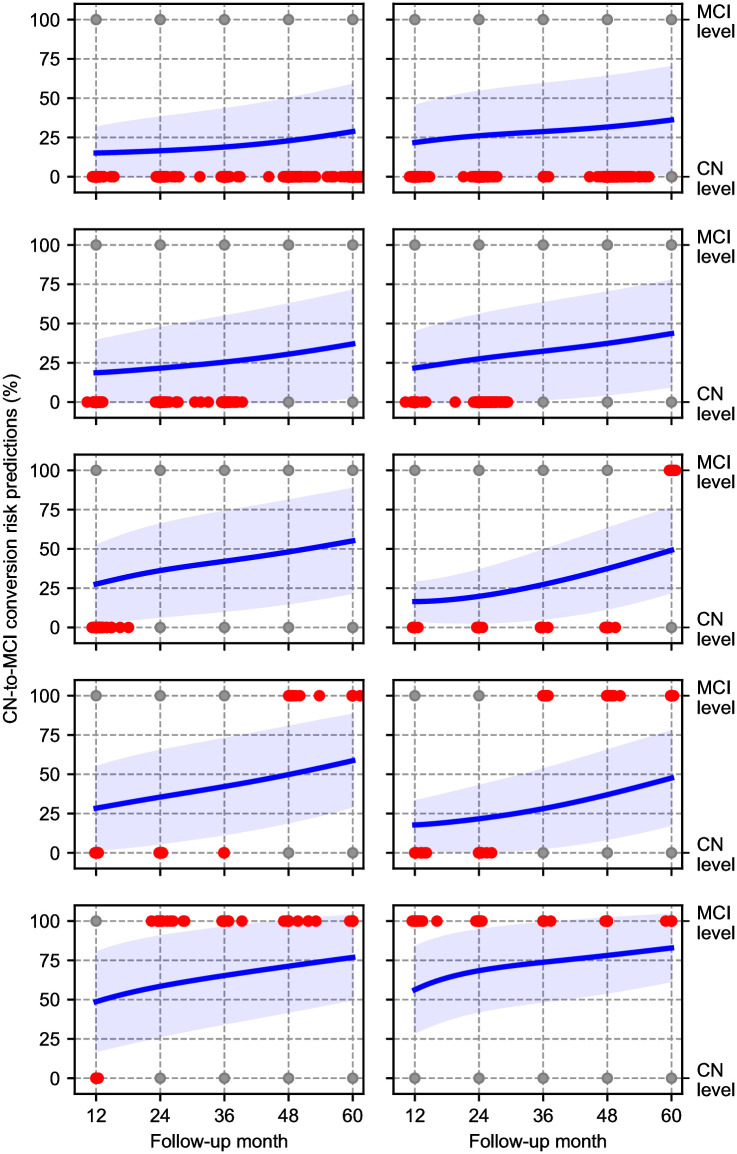
Conversion risk predictions of NMM for CN baseline participants with different ground truth disease trajectories. Blue line is the average MCI conversion risk with 68% confidence. Red dots represent the observed diagnosis time (*x*-coordinate) and the observed diagnosis (*y*-coordinate) of the participants with the corresponding trajectory. Grey dots are for reference.

**Fig 6:**
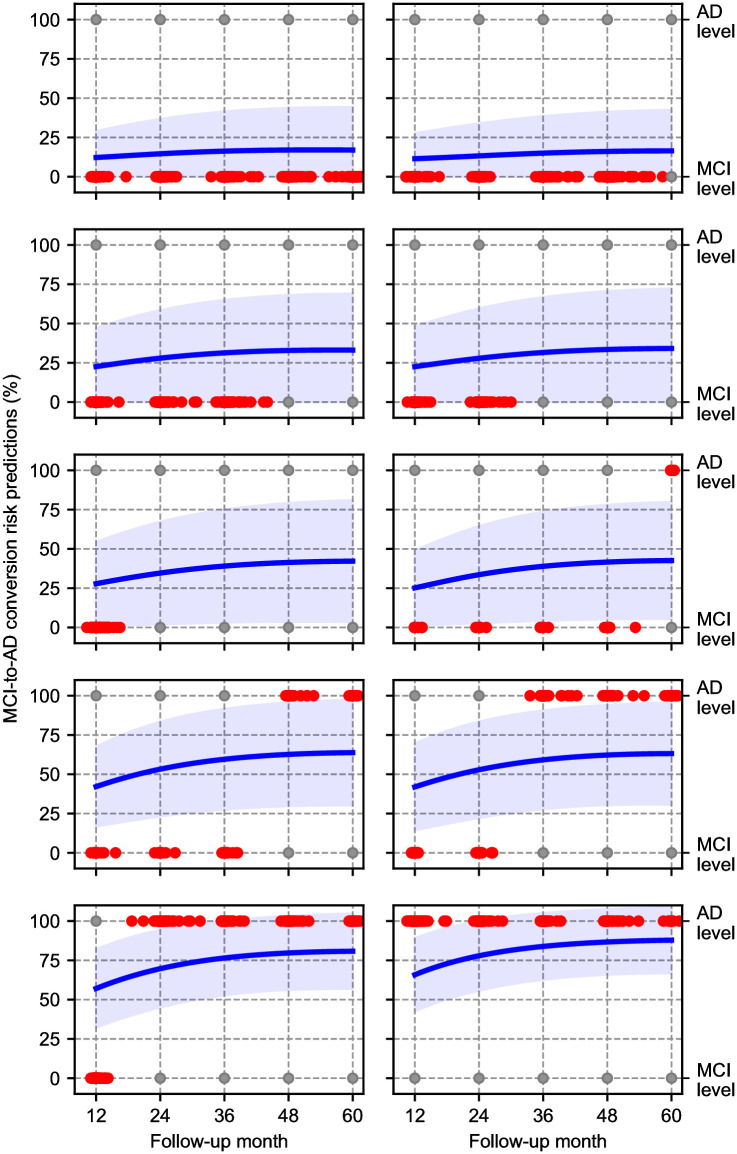
Conversion risk predictions of NMM for MCI baseline participants with different ground truth disease trajectories. Blue line is the average AD conversion risk with 68% confidence. Red dots represent the observed diagnosis time (*x*-coordinate) and the observed diagnosis (*y*-coordinate) of the participants with the corresponding trajectory. Grey dots are for reference.

The conversion risk predictions that we show in Figs 5 and 6 suggest that NMM captures the continuous disease dynamics. However, NMM’s predictions are not always exactly aligned with the timing of events. This issue can be related to various biases in subject recruitment and follow-up in the ADNI [61]. For example, the data suffer from a “temporal bias” [62] that is caused by the fact that baseline visits are not distributed uniformly over latent disease stages. These shortcomings require further investigations, likely demanding novel methodological approaches that can address the selection and temporal biases in the data and possibly exploiting other cohorts, as in [63].

## Conclusion

We have presented a machine learning approach that uses participants’ multimodal baseline data with arbitrary missingness, to predict their future diagnostic status at any time point. We have demonstrated that our model can capture disease progression dynamics and produce future conversion predictions that are highly accurate. Our analyses allow us to dissect the impact of modeling choices and input data types. We found that molecular biomarkers are more useful for predicting MCI-to-AD conversion than CN-to-MCI conversion. Our results show that MRI features are essential for both types of predictions, yet different types of MRI-derived measurements can be useful in different stages.

## Supporting information

S1 Table Performance of each model in terms of AUC ROC for CN baseline participants. Data format is mean ± standard error. LSM^†^ is a standard linear ridge regression model that is an alternative implementation of LSM (Linear Single-year Model). NMM^†^ is a slight modification of NMM (Nonlinear Multi-year Model), where the input time-to-follow-up (Δt) feature is encoded as the closest annual visit time. NSM: Nonlinear Single-year Model.
(PDF)

S2 Table Performance of each model in terms of AUC ROC for MCI baseline participants. See caption of S1 Table.
(PDF)

S1 Fig Predictive performance of NMMs with different architectures. ROC AUC values are averaged across 200 80–20 data splits. Error bars indicate the standard error across these splits. NMM, Nonlinear Multi-year Model with the architecture shown in Fig 1; NMM (3-layer), Nonlinear Multi-year Model with a three-layer architecture; NMM (Optimized), Nonlinear Multi-year Model with optimized architectures for each test set. Details of NMM (3-layer) and NMM(optimized) can be found in S1 Text.
(PDF)

S1 Text Details of NMM (3-layer) and NMM (optimized). We use the same hyperparameters and activation functions for NMM (3-layer) and NMM (optimized) as NMM. NMM (3-layer) has an architecture consisting of 2 hidden layers with a width of 128 and an output layer of width 3. NMM (optimized) architectures for each test split are searched over a 3 × 3 grid, characterized by two parameters: width (W) and depth (D). W represents the number of neurons in the first hidden layer, and it can be either 64, 128, or 256. D represents the depth of the architecture in terms of equally wide blocks in Fig 1, i.e., a D of 1 means the architecture has 3 hidden layers of width W; a D of 2 means the architecture has 3 hidden layers of width W, followed by 5 hidden layers of width W/2; and a depth of 3 means the architecture has 3 hidden layers of width W, followed by 5 hidden layers of width W/2, followed by 2 hidden layers of width W/4. All architectures have an output layer with a width of 3. The best architecture is chosen by monitoring the validation loss in one of the train/validation splits.
(PDF)
