# Supplementary material for: Machine learning based multi-modal prediction of future decline toward Alzheimer’s disease: An empirical study
Source: PLoS One. 2022 Nov 16;17(11):e0277322. doi: 10.1371/journal.pone.0277322 (PMC9668188; doi:10.1371/journal.pone.0277322)
Supplement: S1 Table — Data format is mean ± standard error. LSM† is a standard linear ridge regression model that is an alternative implementation of LSM (Linear Single-year Model). NMM† is a slight modification of NMM (Nonlinear Multi-year Model), where the input time-to-follow-up (Δt) feature is encoded as the closest annual visit time. NSM: Nonlinear Single-year Model. (PDF) [file pone.0277322.s001.pdf]

S1 Table. Performance of each model in terms of AUC ROC for CN baseline participants. Data format is mean ± standard error. LSM† is a standard linear ridge regression model that is an alternative implementation of LSM (Linear Single-year Model). NMM† is a slight modification of NMM (Nonlinear Multi-year Model), where the input time-to-follow-up (Δt) feature is encoded as the closest annual visit time. NSM: Nonlinear Single-year Model.

Follow-up year	CN baseline				
	LSM†	LSM	NSM	NMM	NMM†
1	80.66 ± 0.72	83.88 ± 0.68	88.73 ± 0.45	90.4 ± 0.32	89.94 ± 0.33
2	78.45 ± 0.47	79.41 ± 0.50	84.32 ± 0.47	86.28 ± 0.34	86.37 ± 0.33
3	80.83 ± 0.41	81.99 ± 0.38	85.24 ± 0.34	85.78 ± 0.35	85.72 ± 0.35
4	77.39 ± 0.49	78.66 ± 0.42	81.07 ± 0.38	81.98 ± 0.39	82.08 ± 0.38
5	80.84 ± 0.43	81.22 ± 0.42	82.10 ± 0.39	84.08 ± 0.38	84.23 ± 0.37
