# Supplementary material for: Machine learning based multi-modal prediction of future decline toward Alzheimer’s disease: An empirical study
Source: PLoS One. 2022 Nov 16;17(11):e0277322. doi: 10.1371/journal.pone.0277322 (PMC9668188; doi:10.1371/journal.pone.0277322)
Supplement: S2 Table — See caption of S1 Table. (PDF) [file pone.0277322.s002.pdf]

S2 Table. Performance of each model in terms of AUC ROC for MCI baseline participants.
See caption of S1 Table.

Follow-up year	MCI baseline				
	LSM†	LSM	NSM	NMM	NMM†
1	82.11 ± 0.29	83.67 ± 0.27	83.88 ± 0.25	85.42 ± 0.23	85.47 ± 0.23
2	86.49 ± 0.20	87.74 ± 0.18	87.79 ± 0.18	88.51 ± 0.16	88.52 ± 0.16
3	89.32 ± 0.18	90.22 ± 0.15	89.82 ± 0.16	89.93 ± 0.14	89.98 ± 0.14
4	92.11 ± 0.16	93.03 ± 0.14	93.21 ± 0.14	93.32 ± 0.14	93.31 ± 0.13
5	91.45 ± 0.18	92.44 ± 0.16	92.68 ± 0.16	92.84 ± 0.15	92.87 ± 0.16
