# Supplementary figures and images for: Machine learning based multi-modal prediction of future decline toward Alzheimer’s disease: An empirical study
Source: PLoS One. 2022 Nov 16;17(11):e0277322. doi: 10.1371/journal.pone.0277322 (PMC9668188; doi:10.1371/journal.pone.0277322)

CN-to-MCI conversion

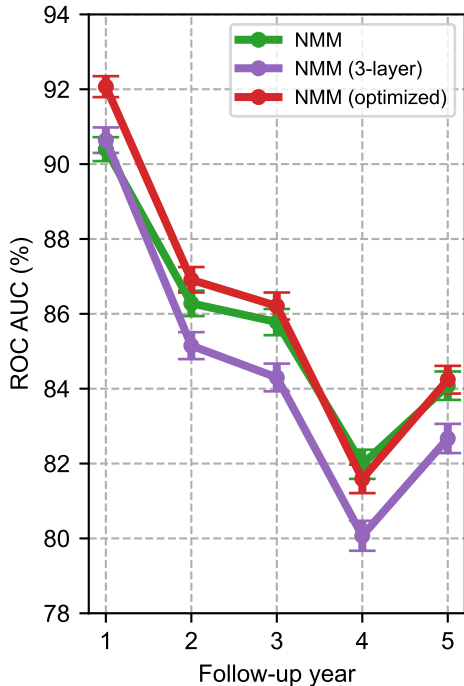

MCI-to-AD conversion

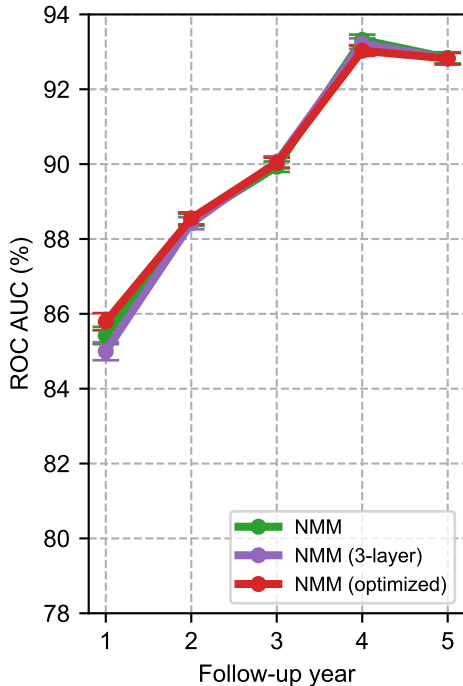

Supplement: S1 Fig — ROC AUC values are averaged across 200 80–20 data splits. Error bars indicate the standard error across these splits. NMM, Nonlinear Multi-year Model with the architecture shown in Fig 1; NMM (3-layer), Nonlinear Multi-year Model with a three-layer architecture; NMM (Optimized), Nonlinear Multi-year Model with optimized architectures for each test set. Details of NMM (3-layer) and NMM(optimized) can be found in S1 Text. (PDF) [file pone.0277322.s003.pdf]
