# Supplementary material for: Machine learning based multi-modal prediction of future decline toward Alzheimer’s disease: An empirical study
Source: PLoS One. 2022 Nov 16;17(11):e0277322. doi: 10.1371/journal.pone.0277322 (PMC9668188; doi:10.1371/journal.pone.0277322)
Supplement: S1 Text — We use the same hyperparameters and activation functions for NMM (3-layer) and NMM (optimized) as NMM. NMM (3-layer) has an architecture consisting of 2 hidden layers with a width of 128 and an output layer of width 3. NMM (optimized) architectures for each test split are searched over a 3 × 3 grid, characterized by two parameters: width (W) and depth (D). W represents the number of neurons in the first hidden layer, and it can be either 64, 128, or 256. D represents the depth of the architecture in terms of equally wide blocks in Fig 1, i.e., a D of 1 means the architecture has 3 hidden layers of width W; a D of 2 means the architecture has 3 hidden layers of width W, followed by 5 hidden layers of width W/2; and a depth of 3 means the architecture has 3 hidden layers of width W, followed by 5 hidden layers of width W/2, followed by 2 hidden layers of width W/4. All architectures have an output layer with a width of 3. The best architecture is chosen by monitoring the validation loss in one of the train/validation splits. (PDF) [file pone.0277322.s004.pdf]

S1 Text. Details of NMM (3-layer) and NMM (optimized). We use the same hyperparameters and activation functions for NMM (3-layer) and NMM (optimized) as NMM. NMM (3-layer) has an architecture consisting of 2 hidden layers with a width of 128 and an output layer of width 3. NMM (optimized) architectures for each test split are searched over a 3×3 grid, characterized by two parameters: width (W) and depth (D). W represents the number of neurons in the first hidden layer, and it can be either 64, 128, or 256. D represents the depth of the architecture in terms of equally wide blocks in Fig 1, i.e., a D of 1 means the architecture has 3 hidden layers of width W; a D of 2 means the architecture has 3 hidden layers of width W, followed by 5 hidden layers of width W/2; and a depth of 3 means the architecture has 3 hidden layers of width W, followed by 5 hidden layers of width W/2, followed by 2 hidden layers of width W/4. All architectures have an output layer with a width of 3. The best architecture is chosen by monitoring the validation loss in one of the train/validation splits.
